# Mitochondrial Fission Induces Glycolytic Reprogramming in Cancer-Associated Myofibroblasts, Driving Stromal Lactate Production, and Early Tumor Growth

**DOI:** 10.18632/oncotarget.574

**Published:** 2012-08-07

**Authors:** Carmela Guido, Diana Whitaker-Menezes, Zhao Lin, Richard G. Pestell, Anthony Howell, Teresa A. Zimmers, Mathew C. Casimiro, Saveria Aquila, Sebastiano Ando’, Ubaldo E. Martinez-Outschoorn, Federica Sotgia, Michael P. Lisanti

**Affiliations:** ^1^ The Jefferson Stem Cell Biology and Regenerative Medicine Center, Kimmel Cancer Center, Thomas Jefferson University, Philadelphia, PA; ^2^ Departments of Stem Cell Biology & Regenerative Medicine, and Cancer Biology, Kimmel Cancer Center, Thomas Jefferson University, Philadelphia, PA; ^3^ Department of Pharmaco-Biology, and Faculty of Pharmacy, University of Calabria, Arcavacata di Rende, Cosenza, Italy; ^4^ Department of Medical Oncology, Kimmel Cancer Center, Thomas Jefferson University, Philadelphia, PA; ^5^ Manchester Breast Centre & Breakthrough Breast Cancer Research Unit, Paterson Institute for Cancer Research; School of Cancer, Enabling Sciences and Technology, Manchester Academic Health Science Centre, University of Manchester, UK

**Keywords:** mitochondrial fission, myofibroblast, oxidative stress, tumor stroma, cancer associated fibroblast, aerobic glycolysis, autophagy, mitophagy, cancer metabolism, tumor initiation, onco-catabolite

## Abstract

Recent studies have suggested that cancer cells behave as metabolic parasites, by inducing oxidative stress in adjacent normal fibroblasts. More specifically, oncogenic mutations in cancer cells lead to ROS production and the “secretion” of hydrogen peroxide species. Oxidative stress in stromal fibroblasts then induces their metabolic conversion into cancer-associated fibroblasts. Such oxidative stress drives the onset of autophagy, mitophagy, and aerobic glycolysis in fibroblasts, resulting in the local production of high-energy mitochondrial fuels (such as L-lactate, ketone bodies, and glutamine). These recycled nutrients are then transferred to cancer cells, where they are efficiently burned via oxidative mitochondrial metabolism (OXPHOS). We have termed this new energy-transfer mechanism “Two-Compartment Tumor Metabolism”, to reflect that the production and consumption of nutrients (L-lactate and other catabolites) is highly compartmentalized. Thus, high-energy onco-catabolites are produced by the tumor stroma.

Here, we used a genetic approach to stringently test this energy-transfer hypothesis. First, we generated hTERT-immortalized fibroblasts which were genetically re-programmed towards catabolic metabolism. Metabolic re-programming towards glycolytic metabolism was achieved by the recombinant over-expression of MFF (mitochondrial fission factor). MFF over-expression results in extensive mitochondrial fragmentation, driving mitochondrial dysfunction. Our results directly show that MFF-fibroblasts undergo oxidative stress, with increased ROS production, and the onset of autophagy and mitophagy, both catabolic processes. Mechanistically, oxidative stress induces autophagy via NF-kB activation, also providing a link with inflammation. As a consequence MFF-fibroblasts showed intracellular ATP depletion and the extracellular secretion of L-lactate, a critical onco-catabolite. MFF-fibroblasts also showed signs of myofibroblast differentiation, with the expression of SMA and calponin.

Importantly, MFF-fibroblasts signficantly promoted early tumor growth (up to 6.5-fold), despite a 20% overall reduction in angiogenesis. Thus, catabolic metabolism in cancer-associated fibroblasts may be a critical event during tumor intiation, allowing accelerated tumor growth, especially prior to the onset of neo-angiogenesis.

## INTRODUCTION

Until recently, the “Warburg Effect” was considered the main metabolic mechanism through which cancer cells acquire their energy, for promoting tumor progression and metastasis [1-3]. More specifically, this model emphasizes that tumor cells develop altered mitochondrial metabolism, resulting in inefficient aerobic respiration and a switch towards glycolytic metabolism, with the increased conversion of glucose to L-lactate[1-3]. However, several key studies have shown that tumor cell lines can maintain normal or even elevated mitochondrial function, suggesting that there might be another explanation for increased aerobic glycolysis in human tumors [4-7].

In particular, over the last 10 years, it has been recognized that the tumor microenvironment is intimately involved in tumor development and progression [8-11]. Both in vitro and in vivo studies have now provided convincing evidence that “activated” stromal fibroblasts, a.k.a., myofibroblasts, may play a critical role in initiating tumor recurrence, via paracrine interactions with adjacent tumor epithelial cells [12-19].

Interestingly, recent experimental evidence indicates that cancer-associated fibroblasts have a catabolic phenotype, and undergo autophagy and mitophagy, resulting in the onset of glycolytic metabolism, driving L-lactate production, and its release into the tumor microenvironment. [16, 20-48]. Then, L-lactate functions as an onco-metabolite, stimulating mitochondrial biogenesis and OXPHOS in adjacent cancer cells, directly providing energy for tumor growth [34, 43, 44]. In this context, stromal L-lactate serves as a high-energy mitochondrial “fuel” for cancer cells. We have termed this new model of cancer metabolism “Two-Compartment Tumor Metabolism”, where two opposing metabolic compartments co-exist, side-by-side, with stromal glycolysis fueling OXPHOS in cancer cells [34, 43, 44].

A key prediction of this hypothesis, also known as the “Reverse Warburg Effect”, is that catabolic fibroblasts should promote tumor growth, without any increases in angiogenesis [41, 42, 48]. To test this hypothesis more directly, we used a genetic approach to create stromal fibroblasts with mitochondrial dysfunction. For this purpose, we stably over-expressed MFF (mitochondrial fission factor) in an hTERT-immortalized human fibroblast cell line and studied their properties. MFF is a novel protein identified as a component of the mitochondrial membrane, which is required for mitochondrial fission [49]. Conversely, MFF over-expression leads to mitochondrial fragmentation and dysfunction.

Here, we show that MFF over-expressing fibroblasts undergo oxidative stress, with increased ROS production, and NF-kB activation, driving the onset of autophagy, mitophagy, and, ultimately, glycolytic metabolism. As a consequence of this mitochondrial dysfunction, MFF-fibroblasts show evidence of ATP depletion, and increased L-lactate production, especially under conditions of hypoxic stress. Most importantly, MFF over-expression in stromal fibroblasts is sufficient to drive the onset of myofibroblastic differentiation and to effectively promote early tumor growth, without any increases in neo-angiogenesis.

## RESULTS

### MFF over-expression in fibroblasts induces mitochondrial dysfunction

**Figure 1:**
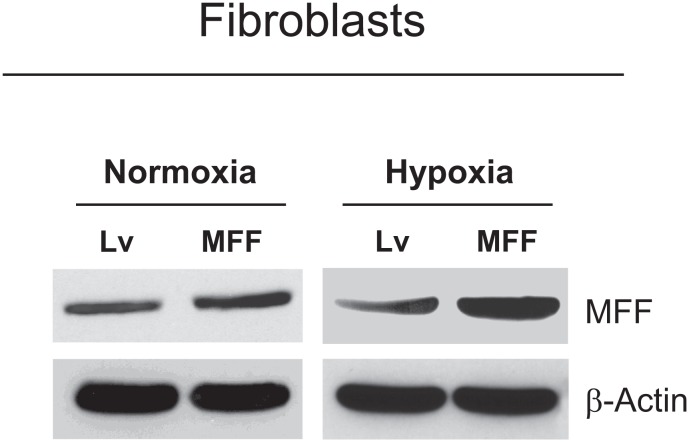
Generation of fibroblasts over-expressing MFF Fibroblasts over-expressing MFF were generated via lenti-viral transduction. Empty vector (Lv) control cells were generated in parallel. Fibroblasts were maintained under conditions of normoxia or hypoxia for 6 hours. MFF protein expression levels were evaluated by immuno-blotting. β-actin was used as an equal protein loading control.

Mitochondrial Fission Factor (MFF) is involved in the fission process normally required to maintain the physiologic function of mitochondria [49]. However, it remains unknown if MFF over-expression plays a role in the breast cancer tumor stroma. Thus, we stably expressed MFF in hTERT-immortalized human fibroblasts. Figure 1 shows that we successfully over-expressed MFF in fibroblasts, and this is especially evident upon exposure to hypoxic stress.

**Figure 2:**
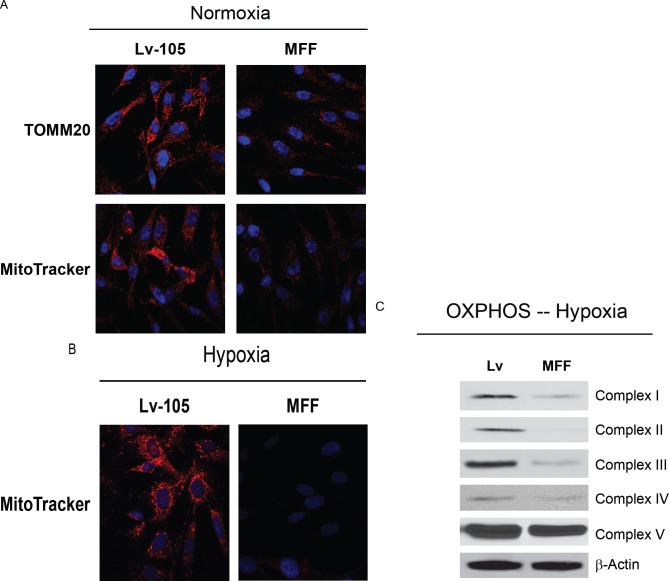
Fibroblasts over-expressing MFF show mitochondrial dysfunction (A) *Normoxia*. To determine mitochondrial mass, cells were immuno-stained with TOMM20, a mitochondrial membrane marker. Note that TOMM20 is significantly decreased in MFF-fibroblasts, suggesting that MFF reduces mitochondrial mass. To determine functional mitochondrial activity, cells were independently labeled with MitoTracker. Note the strong reduction in mitochondrial membrane potential. Original magnification, 40X. (B) *Hypoxia*. MitoTracker staining was performed in control (Lv-105) and MFF-fibroblasts kept under hypoxia for 6 hours (0.5% O_2_). Note that MFF induces a very strong reduction in mitochondrial membrane potential, particularly upon hypoxic stress. Original magnification, 40X. (C) OXPHOS protein levels were evaluated by immuno-blotting after 6 hours of hypoxia. Note that MFF-fibroblasts display a strong down-regulation of key components of complex I (20 kDa subunit), II (30 kDa subunit), III (Core-2), and IV (COX-II). β-actin was used as an equal protein loading control.

To evaluate if increased mitochondrial fission alters the bioenergetic state of mitochondria, we first evaluated mitochondrial mass by immuno-staining with antibodies directed against TOMM20, a mitochondrial outer membrane marker. Figure 2A shows that TOMM20 immuno-staining is reduced in fibroblasts over-expressing MFF, relative to controls.

Next, we used MitoTracker to assess the status of mitochondrial activity. Interestingly, relative to control cells, MFF-fibroblasts display a strong reduction in MitoTracker staining, both under normoxic (Figures 2A) and hypoxic conditions (Figures 2B), indicative of decreased mitochondrial membrane potential. Especially under hypoxia, we observed a more pronounced loss of mitochondrial function. To independently validate these data, we evaluated the expression of OXPHOS components upon hypoxic stress. Mitochondrial components of complexes I, II, III, and IV were all dramatically reduced in fibroblasts over-expressing MFF, relative to empty vector controls (Figure 2C).

**Figure 3:**
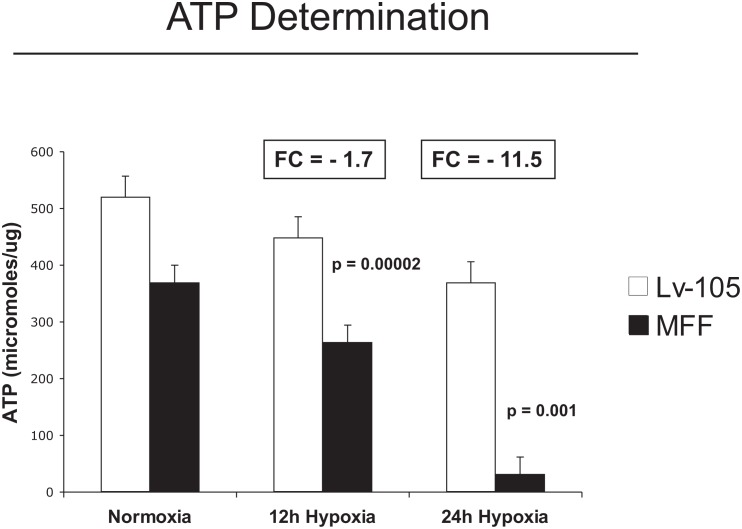
Fibroblasts over-expressing MFF show decreased ATP levels Intracellular ATP content was measured using empty vector control (Lv-105) and MFF-fibroblasts maintained under normoxia or hypoxic stress (for 12 and 24 hours). Note that as compared to control cells, MFF-fibroblasts display a 1.7-fold and a 11.5-fold reduction in steady-statet ATP leves, respectively, after 12 and 24 hours of hypoxia. These results suggest that the mitochondrial dysfunction in MFF over-expressing fibroblasts is associated dramatic decreases in intracellular ATP.

An imbalance between fusion and fission can cause mitochondrial dysfunction, with decreased ATP production. Thus, we evaluated the steady-state ATP levels in MFF-fibroblasts and empty vector controls under hypoxia. Figure 3 shows that after 12 and 24 hours of hypoxia, MFF-fibroblasts display a 1.7-fold and a 11.5-fold reduction, respectively, in ATP levels, indicating that mitochondrial dysfunction is associated with decrease intracellular ATP, as predicted.

Taken together, these data demonstrate that, as reported in the literature, an increase in mitochondrial fission drives mitochondrial fragmentation associated with mitochondrial dysfunction, decreased membrane potential, decreased oxidative phosphorylation, and reduced ATP levels.

### Fibroblasts over-expressing MFF display metabolic remodeling, with a shift towards glycolytic metabolism

**Figure 4:**
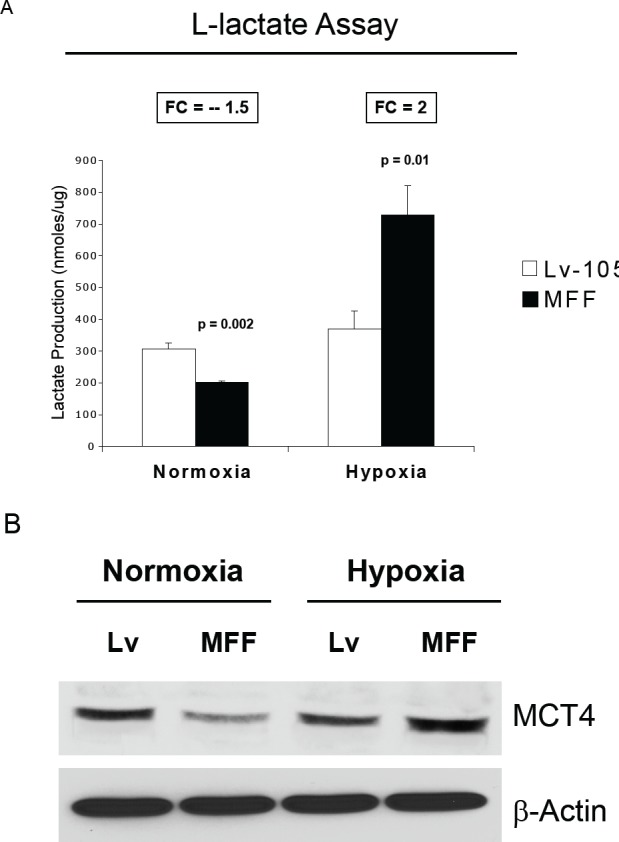
Fibroblasts over-expressing MFF show increased L-lactate generation and MCT4 expression, under hypoxia (A) Empty vector (Lv-105) and MFF-fibroblasts were grown under normoxic or hypoxic conditions (6 hours), and then subjected to biochemical analysis, to determine L-lactate content in the tissue culture media. The graph shows that MFF-fibroblasts show a 1.5-fold decrease in L-lactate production under normoxia. However, when challenged with hypoxia, MFF-fibroblasts exhibit a 2-fold increase in L-Lactate generation, as compared with empty vector (Lv-105) control cells. (B) MCT4 is a transporter which mediates the efflux of L-lactate and is a marker of oxidative stress and aerobic glycolysis. Empty vector and MFF-fibroblasts were maintained under normoxia or hypoxia (6 hours), and were subjected to immuno-blotting with anti-MCT4 antibodies. Consistent with the L-lactate assay results, MCT4 levels are down-regulated in MFF-fibroblasts under normoxia; in contrast, MCT4 levels are strongly up-regulated in MFF-fibroblasts under hypoxia.

Decreased mitochondrial functional activity is associated with metabolic changes, with a shift towards glycolysis. Thus, we next examined the accumulation of L-lactate in the conditioned media of MFF-fibroblasts versus control fibroblasts. Figure 4A shows that under hypoxic conditions, MFF-fibroblasts display a 2-fold increase in L-lactate secretion, relative to control cells. Interestingly, however, in normoxia, MFF-fibroblasts show a 1.5-fold decrease in lactate generation, likely due to compensatory adaptation. Thus, MFF promotes a glycolytic phenotype under conditions of hypoxia that are usually found within tumors in vivo.

To validate that MFF-fibroblasts are more glycolytic, we performed a immuno-blot analysis with antibodies directed against MCT4. MCT4 is the transporter that extrudes L-lactate from glycolytic cells. MCT4 is also HIF-1a target and is an hypoxia-inducible gene. Consistent with the results of the lactate assay, Figure 4B shows that during conditions of hypoxic stress, MCT4 expression is upregulated in MFF-fibroblasts, as compared to control cells. In normoxia, however, MCT4 levels are decreased in MFF-fibroblasts, as compared to control cells. These results suggest that under the low oxygen tension conditions found in human tumors, MFF-fibroblasts are more glycolytic and display an efflux of high-energy mitochondrial fuels (such as L-lactate) into the extracellular microenvironment.

### Fibroblasts over-expressing MFF show increased oxidative stress and autophagy, with NFkB-hyperactivation

**Figure 5:**
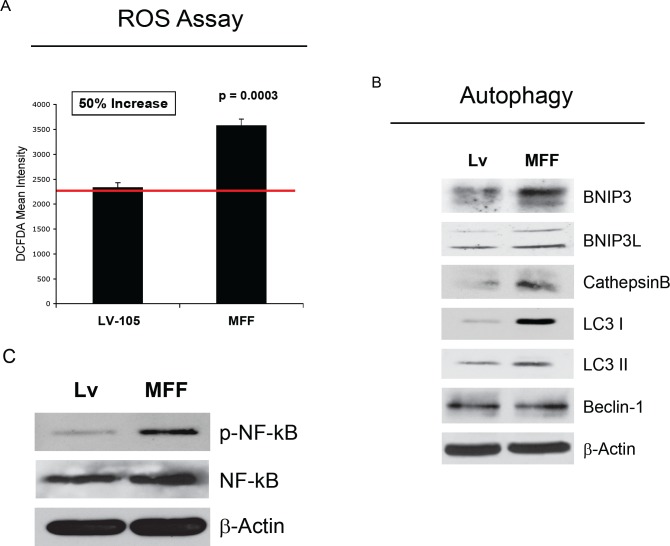
Fibroblasts over-expressing MFF show increased ROS generation, and induction of autophagy, with NF-kB hyper-activation (A) ROS levels were measured by FACS analysis, as described under Materials and Methods. Note that MFF over-expression induces a 50% increase in ROS production, as compared to control cells. (B) Induction of an autophagic/mitophagic process was analyzed on cellular extracts of fibroblasts expressing MFF and empty vector controls by immunoblotting. Note that MFF-fibroblasts show the up-regulation of several markers of autophagy (LC3I/II and cathepsin B), and mitophagy (BNIP3 and BNIP3L), as compared to control cells. No changes were observed for Beclin-1 expression. β-actin is shown as an equal loading control. (C) NF-kB activation was monitored by immuno-blotting with phospho-NF-kB antibodies. Note that MFF-fibroblasts show constitutive NF-kB activation, relative to control cells, suggesting that NF-kB activation maybe the mechanism driving the induction of autophagy. β-actin was used as an equal protein loading control.

It is well known that mitochondrial dysfunction induces oxidative stress and that oxidative stress promotes autophagy, driving the removal of damaged mitochondria, via mitophagy. To evaluate if MFF causes increased oxidative stress in stromal cells, we examined the status of ROS production in fibroblasts carrying the Lv-105 empty vector or MFF. ROS levels were measured by FACS analysis. Interestingly, MFF-fibroblasts show a 50% increase in ROS production, as compared to control cells (Figure 5A).

Next, the activation of autophagy/mitophagy was analyzed on protein extracts of fibroblasts harboring MFF by immuno-blotting. Interestingly, MFF induces an autophagy/mitophagy program in fibroblasts, as judged by the upregulation of markers of autophagy (LC3 I, LC3II and cathepsin B), and mitophagy (BNIP3 and BNIP3L) (Figure 5B). Beclin-1 expression remains unchanged. To gain insights into the mechanism(s) triggering autophagy, we monitored the activation the NF-kB pathway by immuno-blotting. Figure 5C shows that MFF-fibroblasts display the constitutive activation of NF-kB, suggesting that NF-kB activation is likely one of the mechanism(s) driving autophagy.

**Figure 6:**
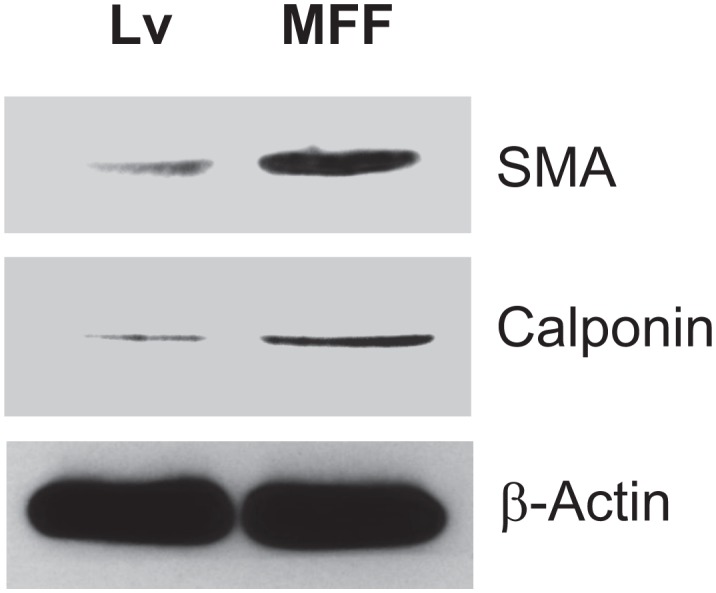
Fibroblasts over-expressing MFF show myofibroblastic features To evaluate if MFF-fibroblasts acquire myofibroblastic features, cells were analyzed by immuno-blotting with antibodies directed against α-SMA and calponin. Note that MFF-fibroblasts show increased expression of two myo-fibroblast markers, namely α-SMA and calponin, indicating that MFF promotes a myofibroblastic differentiation. β-actin was used as an equal protein loading control.

To further characterize the features of MFF-expressing fibroblasts, we investigated if MFF induces a myofibroblastic phenotype. Figure 6 shows that MFF-fibroblasts display the up-regulation of two myofibroblast markers, namely α-SMA and calponin.

### Fibroblasts over-expressing MFF promote tumor growth, especially in the early phases of tumorigenesis, without any increase in neo-angiogenesis

**Figure 7:**
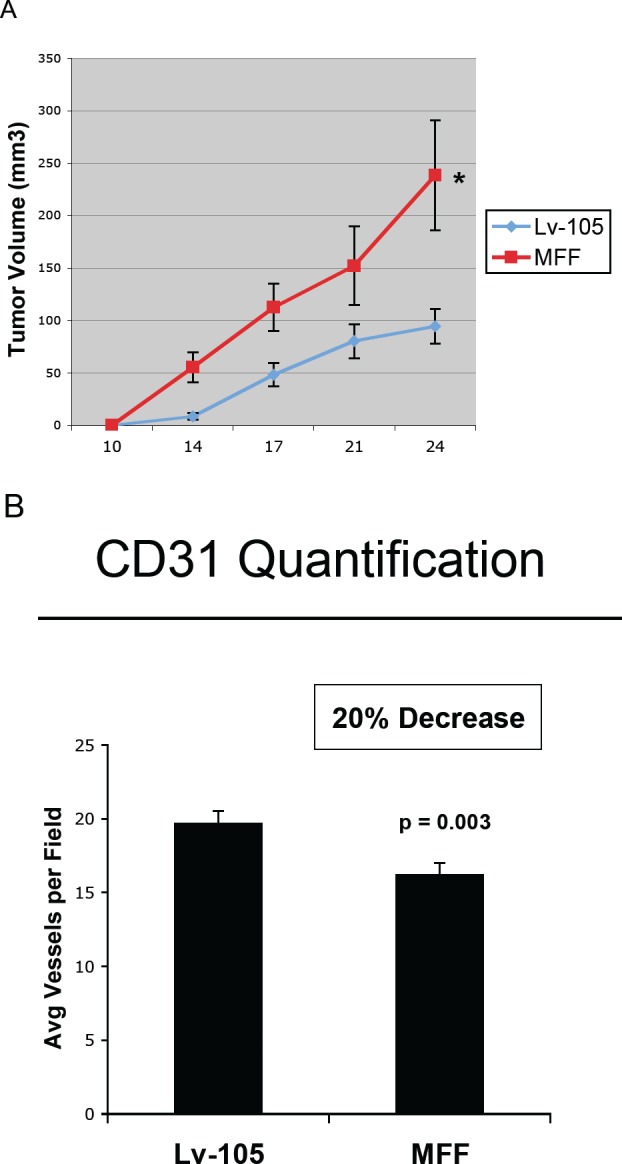
Fibroblasts over-expressing MFF promote tumor growth, independently of neo-angiogenesis (A) To assess the functional in vivo effects of MFF over-expression in fibroblasts, we co-injected MFF-fibroblasts or vector alone control fibroblasts with MDA-MB-231 breast cancer cells into the flanks of nude mice. Then, tumor growth rates were monitored every 3-4 days for 24 days. Note that MFF-fibroblasts promote a signficant increase in tumor growth rates, as compared with control fibroblasts. N = 10 tumors per experimental group. (B) Tumor angiogenesis was monitored by CD31 immuno-staining on tumor frozen sections. Note that MFF-xenografts show a 20% decrease in tumor neo-vascularization, as compared to controls. These data indicate that the MFF-induced tumor growth is independent of angiogenesis.

To evaluate the effects of MFF on tumor development *in vivo*, MFF-fibroblasts or empty vector control fibroblasts were co-injected with MDA-MB-231 human breast cancer cells into the flanks of nude mice. *Interestingly, Figure 7A and Table 1 demonstrate that* MFF over-expression in fibroblasts increases tumor growth rates (up to 6.5-fold), especially in the early phases of tumor development, suggesting that MFF can act as a stromal tumor promoter.

**Table 1:** MFF over-expressing fibroblasts increase tumor growth, especially during the early phase of tumorigenesis Note that fibroblasts over-expressing MFF greatly increase tumor growth. Fold increases in tumor growth rates for MDA-MB-231 xenografts are shown (co-injected with MFF over-expressing fibroblasts *versus* empty vector controls (Lv-105)). Interestingly, the role of MFF is most relevant in the early phase of tumor development, suggesting that increased mitochondrial fission in the stroma can metabolically promote and support tumor initiation.

Days Post-Injection	Lv-105 Tumor Volume	MFF Tumor Volume	Fold- Increase (MFF/control)	p-values
14	8.51	55.37	6.5	0.003
17	48.14	112.42	2.3	0.01
21	80.40	152.32	1.9	0.047
24	94.38	238.42	2.5	0.009

To evaluate if the MFF-tumor promoting effects are dependent on increased angiogenesis, tumor xenografts were immunostained with antibodies against CD31, an endothelial cell marker. CD31 quantification indicates that the tumors derived from MFF fibroblasts display a 20% decrease in vessel density, as compared to the control tumors, suggesting that increased angiogenesis is not the mechanism promoting tumor growth.

## DISCUSSION

Mitochondria play a critical role in all the normal physiological events that maintain organismal energy homeostasis or balance [50-54]. As a consequence, mitochondrial dysregulation is likely the “root cause” of several human disease(s), and especially epithelial cancers [50, 51, 55, 56].

A new hypothesis is that cancer is not a cell autonomous disease, but rather a disease of the tumor microenvironment [32-34, 41] [43, 44]. We have proposed that cancer cells “fertilize” the tumor microenvironment, via the secretion of hydrogen peroxide [25, 29, 57]. Oxidative stress in the tumor stroma then provides high-energy recycled nutrients, via the onset of catabolic metabolic processes, such as autophagy, mitophagy, and aerobic glycolysis [32-34, 41] [43, 44]. This localized production of onco-catabolites (such as L-lactate, ketones, and glutamine) then “fuels” the expansion and metastasis of cancer cells, via oxidative mitochondrial metabolism, driving the anabolic growth of tumors [20, 23, 58-60]. A simple prediction of this hypothesis is that catabolic fibroblasts should be able to promote the growth of cancer cells, independently of angiogenesis [37, 38, 61].

Here, we used MFF (mitochondrial fission factor) [49] as a genetic tool to assess the metabolic effects of a “pre-fertilized” microenvironment on tumor growth and angiogenesis. MFF is a novel protein that plays a key role in regulating the overall size and, hence, the activity mitochondria [49]. MFF forms a tight complex with the GTPase, dynamin-related protein (Drp1), and activates the mitochondrial fission process, causing the extensive fragmentation of mitochondria [49]. Mitochondrial fission is involved in mitochondrial turn-over by autophagy (mitophagy) and is associated with decreased mitochondrial membrane potential and low levels of ATP [62, 63].

hTERT-immortalized human fibroblasts stably over-expressing MFF were generated using a lentiviral vector approach, to evaluate the potential tumor-promoting effects of metabolic remodeling in the tumor microenvironment. Now, we show that MFF-driven mitochondrial fission in stromal fibroblasts is indeed sufficient to induce a catabolic cancer-associated fibroblast phenotype. MFF-fibroblasts showed the up-regulation of myofibroblast markers (SMA and calponin), as well as a shift towards glycolytic metabolism. MFF-fibroblasts underwent constitutive autophagy and mitophagy, due to ROS over-production and NF-kB activation, driving glycolytic metabolism. For example, under conditions of hypoxic stress, MFF fibroblasts showed a >10-fold reduction in steady-state ATP levels, and a 2-fold increase in L-lactate secretion. Thus, increased mitochondrial fission is sufficient to induce oxidative stress in the tumor microenvironment and defects in oxidative mitochondrial metabolism (OXPHOS, as seen by immuno-blot analysis), thereby promoting a catabolic phenotype, which results in the over-production of the onco-metabolite, L-lactate.

As a consequence of oxidative stress and a shift towards glycolytic metabolism, MCT4 was specifically up-regulated by hypoxia in MFF-fibroblasts. Consistent with our current hypothesis, MCT4 is a HIF1 target gene that functions in the export of L-lactate from cells undergoing oxidative stress induced glycolysis, to prevent the intracellular accumulation of L-lactate. Interestingly, we have recently shown that the up-regulation of stromal MCT4 in cancer-associated fibroblasts is a strong predictor of poor clinical outcome and lethality in human breast cancer patients [45, 46, 48, 64]. Conversely, the expression levels of MCT4 in epithelial cancer cells had no prognostic value, indicating that the conventional Warburg effect does not predict clinical outcome [45, 46, 48].

In accordance with our observations on the prognostic value of stromal MCT4, it has been known for many years that elevated serum and tumor-associated L-lactate levels are strong predictors of poor clinical outcome (extensively reviewed in [46]). However, L-lactate production was previously attributed to increased glycolysis in human cancer cells, rather than in cancer-associated fibroblasts [1].

Consistent with the idea that stromal L-lactate production is associated with lethal cancer metabolism, MFF-fibroblasts significantly promoted tumor growth, especially in the early phase of tumorigenesis (~6.5-fold), without any increases in tumor angiogenesis. Rather, tumors grown with MFF-fibroblasts showed a 20% reduction in tumor vascularization, but increased tumor growth. Thus, stromal L-lactate production can drive anabolic tumor growth, as L-lactate functions as both as a i) high-energy mitochondrial fuel, and as a ii) signaling molecule that promotes mitochondrial biogenesis, in human cancer cells [33].

In fact, human breast cancer cells (MCF7 cells, treated with and without L-lactate) were previously used to generate an L-lactate associated gene signature [33]. Importantly, breast cancer patients with this L-lactate-induced gene signature showed an increased risk for recurrence, metastasis, and poor clinical outcome [33]. Thus, when cancer cells use L-lactate as a mitochondrial fuel source, this metabolic phenotype is a predictor of lethal cancer metabolism [33].

In conclusion, MFF-fibroblasts represent a new model system for studying the role of mitochondrial dysfunction and stromal L-lactate production in tumor initiation, progression, and metastasis, independently of tumor angiogenesis. This phenotype was most evident under hypoxic conditions, which is consistent with the angiogenesis-independent nature of our findings.

## MATERIALS AND METHODS

### Cell culture

MDA-MB-231 human breast cancer cells (stably transfected with GFP were a gift from Dr. Fatatis's Laboratory, Drexel University, Philadelphia, PA) and human immortalized fibroblasts (hTERT-BJ1) were maintained in Dulbecco's modified Eagle's medium (DMEM) implemented with 10% fetal bovine serum (FBS), 100 units/mL penicillin, and 100 μg/mL streptomycin, at 37°C in 5%CO_2_. incubator. For hypoxia experiments, the cells were maintained at 37°C with 0.5%O_2_.

### Lentiviral transduction

For lentiviral transduction, vectors (from GeneCopoeia, Inc.) encoding MFF (EX-Z4766-Lv-105) or empty vector control (EX-NEG-Lv-105) were transfected into the 293Ta packaging cells, using the Lenti-Pac HIV Expression Packaging Kit (GeneCopoeia, Inc.), following the manufacturer's instructions. Two days post-transfection, viral supernatants were collected, centrifuged and filtered (0.45 μM Polyethersulfone low protein filter) and added to the target cells (hTERT-BJ1 fibroblasts) in the presence of 5 μg/ml polybrene. HTERT-fibroblasts were then selected with 1.5 μg/ml puromycin.

### Immunoblot analysis

Cells were harvested into lysis buffer (10mM Tris-HCl pH 7.5, 150 mM NaCl, 1% Triton X-100, 60mM octylglucoside), supplemented with protease and phosphatase inhibitors (Roche Applied Science). After rotation at 4°C for 40 minutes, samples were centrifuged 10 min at 13,000x g at 4°C and the supernatants were collected to remove insoluble debris. For NF-kB detection, cells were scraped in RIPA lysis buffer (50 mM Hepes, 2 mM EDTA, 0.1% SDS, 50 mM NaCl, 1% NP40), containing protease and phosphatase inhibitors, sonicated and incubated on ice for 10 minutes. Then, samples were centrifuged 10 min at 13,000x g at 4°C and the supernatants were collected. Protein samples were separated by SDS-PAGE, and transferred to a nitrocellulose membrane. Membranes were blocked with TBS-Tween (20 mM Tris pH 7.6, 150 mM NaCl, and 0.05% Tween-20) supplemented with 1% BSA and 4% nonfat dry milk for 1 hour at room temperature. For phospho-antibodies, the blocking solution contained only 5% BSA in TBS-Tween. The membranes were incubated with primary antibodies for 1 hour at room temperature. The following antibodies were used: MFF (Abcam, ab81127); MCT4 (Sigma-Aldrich, SAB4503555); Smooth Muscle Actin (Dako, M0851); Calponin 1/2/3 (FL-297) (Santa Cruz, sc-28545); BNIP3 (Abcam, ab10433); BNIP3L (Abcam, ab8399); Cathepsin B (FL-339) (Santa Cruz, sc-13985); LC3 (Abcam, ab48395); NF-kB p65 (Cell Signaling, 3034); phospho-NF-kB p65 (Cell Signaling, 3037); Mitoprofile total OXPHOS cocktail (Mitosciences, MS601). Then, blotting membranes were washed, incubated for 30 min at room temperature with horseradish peroxidase-conjugated secondary antibodies (anti-mouse, 1:6.000 dilution (Pierce) or anti-rabbit 1:5.000 (BD Biosciences/Pharmingen). HRP activity was visualized by enhanced chemiluminescent substrate (Thermo Scientific). As internal control, all membranes were subsequently stripped (Restore Western Blot Stripping Solution, Thermo Scientific) and re-probed with anti β-actin antibodies (Sigma-Aldrich, A5441).

### Mitochondrial activity

Fibroblasts were seeded onto glass coverslips in 12-well plates in complete media. After 24 hours, the media was changed to DMEM containing 2% FBS. After 72 hours, cells were incubated with the pre-warmed 25 nM MitoTracker staining solution (CMTMRos, M7510, Invitrogen) for 15 minutes, and washed (3X) in PBS supplemented with calcium and magnesium. Then, cells were fixed with 2% PFA, washed and incubated with DAPI nuclear stain (Invitrogen, D3571). Samples were mounted with Prolong Gold Anti-Fade mounting reagent (Invitrogen, P36930). Images were collected with a Zeiss LSM510 meta confocal system and acquired with a 60X oil objective.

### Immuno-fluorescence

Cells were washed three times in PBS with 0.1 mM CaCl_2_ and 1 mM MgCl_2_ and fixed with 2% PFA. Then, the cells were permeabilized with ice cold methanol for 10 min, incubated with 25 mM NH_4_Cl in PBS for 10 min, washed with PBS, and incubated with primary antibodies (TOMM20, Santa Cruz, sc-17764) for 1 hour a room temperature. Then, cells were washed with IF buffer (PBS, 1% BSA, 0.1% Tween 20), and incubated for 30 min with fluorochrome-conjugated secondary antibodies. Finally, slides were incubated with the nuclear stain DAPI and mounted. Images were collected with a Zeiss LSM510 meta confocal system, using a 40X oil objective.

### ROS Assay

Fibroblasts were seeded at a density of 130,000 per well in 12-well plates in standard media in quadruplicate. ROS levels were evaluated after 48 hours. Briefly, cells were washed and incubated for 15 min at 37°C with 10μM CM-H2DCFDA (Invitrogen). Then, cells were washed with HBSS and placed in standard media for 15 min at 37°C. Cells were then washed, trypsined, resuspended in PBS. The resulting signal was quantified by FACS using the BD LSRII (BD Bioscience). The results were analyzed using FlowJo software (Tree star Inc.).

### Lactate Assay

120,000 cells were plated into 12-well plates in standard media in quadruplicate. After 24 hours, the media was changed to DMEM containing 2% FBS. Two days later, the media was harvested to measure the concentration L-lactate using the EnzyChrom L-Lactate Assay Kit (BioAssay Systems, ECLC-100), according to the manufacturer's instructions. For hypoxia experiments, cells were incubated for 6 hours in a hypoxia workstation (0.5% O_2_), before lactate measurement. Results were normalized to the protein content.

### ATP Determination

100,000 cells were seeded in 24-well plates in complete media in quadruplicate. The day after, cells were incubated in normoxia or in hypoxia for 12 or 24 hours. Then, cells were lysed in 100 μl of extraction buffer (4 mM EDTA and 0.005% Triton X-100). ATP concentrations were determined by using the ATP determination kit (Invitrogen, A22066). Luminescence was read using a luminometer (Biotek Synergy HT), and values were calculated based on an ATP standard curve. Results were normalized to the protein content.

### Animal Studies

All animals were housed and maintained in a barrier facility at the Kimmel Cancer Center at Thomas Jefferson University under National Institutes of Health (NIH) guidelines. Mice were kept on a 12-hour light/dark cycle with ad libitum access to food and water. Studies were approved by the Institutional Animal Care and Use Committee (IACUC) of Thomas Jefferson University. Briefly, MDA-MB-231 cancer cells (1 × 10^6^) admixed with transfected fibroblasts (3 × 10^5^) in 100 μl of sterile PBS, were injected into the flanks of athymic NCr nude mice (NCRNU; Taconic Farms; 6–8 weeks of age). Mice were then sacrificed at 3-4-weeks post-injection; tumors were dissected and fixed with 10% formalin or flash-frozen in liquid nitrogen-cooled isopentane.

### CD31 immuno-histochemistry

Seven micron frozen sections were cut and fixed with 4% paraformaldehyde in PBS for 10 min at 4°C, and washed 3X with PBS. The sections were blocked with 10% rabbit serum and incubated overnight at 4°C with rat monoclonal CD31 antibodies (550274, BD Biosciences, San Jose, CA) at a dilution of 1:200. The sections were then washed and subsequently incubated with biotinylated rabbit anti-rat IgG (Vector Labs, Burlingame, CA), and then with streptavidin-HRP (Dako, Carpinteria, CA). Immunoreactivity was revealed with 3, 3’ diaminobenzidine and sections were then counter-stained with hematoxylin (Vector), dehydrated and mounted.

### Quantification of CD31(+) Vessels

Four tumors were randomly selected from each group and sections were prepared and stained with a CD31 antibody, as described [22, 23]. For each tumor, 8-16 representative fields (0.25 mm^2^ per field) were selected and the numbers of CD31-positive vessels were quantified using an ocular micrometer and a 20x objective lens. Only healthy, non-necrotic tumor areas were evaluated. The numbers of vessels per field were averaged and the overall average per group and standard deviation were calculated. Statistical significance was evaluated using the Student's t-test. p-values lower than 0.05 were considered statistically significant.
